# Release of TGF-β_3_ from Surface-Modified PCL Fiber Mats Triggers a Dose-Dependent Chondrogenic Differentiation of Human Mesenchymal Stromal Cells

**DOI:** 10.3390/pharmaceutics15041303

**Published:** 2023-04-21

**Authors:** Leonie Berten-Schunk, Yvonne Roger, Heike Bunjes, Andrea Hoffmann

**Affiliations:** 1 Technische Universität Braunschweig, Institut für Pharmazeutische Technologie und Biopharmazie, 38106 Braunschweig, Germany; l.berten-schunk@tu-braunschweig.de; 2 Hannover Medical School, Department of Orthopedic Surgery, Graded Implants and Regenerative Strategies, Laboratory of Biomechanics and Biomaterials, 30625 Hannover, Germany; 3 Niedersächsisches Zentrum für Biomedizintechnik, Implantatforschung und Entwicklung (NIFE), 30625 Hannover, Germany; 4 Technische Universität Braunschweig, Zentrum für Pharmaverfahrenstechnik (PVZ), 38106 Braunschweig, Germany

**Keywords:** TGF-β_3_, chondrogenesis, chitosan nanoparticles, release, implant, tissue transition

## Abstract

The design of implants for tissue transitions remains a major scientific challenge. This is due to gradients in characteristics that need to be restored. The rotator cuff in the shoulder, with its direct osteo-tendinous junction (enthesis), is a prime example of such a transition. Our approach towards an optimized implant for entheses is based on electrospun fiber mats of poly(ε-caprolactone) (PCL) as biodegradable scaffold material, loaded with biologically active factors. Chitosan/tripolyphosphate (CS/TPP) nanoparticles were used to load transforming growth factor-β_3_ (TGF-β_3_) with increasing loading concentrations for the regeneration of the cartilage zone within direct entheses. Release experiments were performed, and the concentration of TGF-β_3_ in the release medium was determined by ELISA. Chondrogenic differentiation of human mesenchymal stromal cells (MSCs) was analyzed in the presence of released TGF-β_3_. The amount of released TGF-β_3_ increased with the use of higher loading concentrations. This correlated with larger cell pellets and an increase in chondrogenic marker genes (*SOX9*, *COL2A1*, *COMP*). These data were further supported by an increase in the glycosaminoglycan (GAG)-to-DNA ratio of the cell pellets. The results demonstrate an increase in the total release of TGF-β_3_ by loading higher concentrations to the implant, which led to the desired biological effect.

## 1. Introduction

With the improvement of health and living conditions, the life expectancy rises and the clinical need for therapy for degenerated tissues and organs increases rapidly, with a strong focus on musculoskeletal disorders [1,2]. One major clinical problem is tearing of the rotator cuff within any of the osteo-tendinous junctions of the respective muscles in the shoulder [3,4,5].

The use of cell-containing therapeutic systems for the regenerative treatment of such injuries involves several challenges. Critical aspects regarding the type, isolation or availability of cells, as well as the phenotypic stability, rejection reactions, risk of infection, malignant degenerations and requirements for clinical use and regulatory provisions, can cause high effort and costs. In a previous in vivo study, the regeneration of the entire articular joint surface without cell transplantation was demonstrated. Hence, cell-free approaches entail many advantages, which further include the exact definition of the ingredients of such systems and, thus, a simplified approval procedure [6,7]. Nevertheless, the anticipated mode of action of such cell-free systems needs to be confirmed in vitro or ex vivo.

The regeneration of tissues low in blood vessels and nerves, like cartilage, is still a complex ssue and currently results in scar or fibrotic tissue rather than in healthy functional tissue [8,9,10]. This leads to pain and even decreased functionality of the specific tissue [11]. In recent decades, cartilage regeneration has been the focus of numerous research projects with different approaches involving new biomaterials, stem cells and/or different cytokines. One possible approach is the use of different types of artificially produced scaffolds, which form the basis for the regeneration of tissues [12,13,14,15]. In various systems, growth factors are introduced into such scaffolds to promote migration, attachment and differentiation of stem cells [16,17,18]. However, none of these approaches was successful in the regeneration of functional cartilage, especially of hyaline cartilage, as in joints. Contrary to joint cartilage, the direct osteo-tendinous junctions of our bodies include a zone of fibrocartilage, which connects tendon to bone. Considering the fibrotic outcomes of many approaches, it might be more realistic to restore such a cartilage zone for biomedical purposes.

The transforming growth factor β (TGF-β) superfamily of multifunctional proteins consists of a variety of different proteins, such as bone morphogenetic protein 2 (BMP-2) and TGF-β isoforms, which are involved in many different processes such as growth, development and differentiation. While BMP-2 is known to induce osteogenesis, the TGF-β isoforms, especially TGF-β_3_, are one of the key players in the chondrogenic differentiation of mesenchymal stromal cells (MSCs) [19,20].

The current study contributes to the development of an innovative implant for the treatment of tears within tissue transitions in the rotator cuff. This implant is meant to be biodegradable and graded regarding various characteristics. The implant will be designed as a scaffold of PCL fibers [21], loaded with proteins. The proteins are selected based on their biological activity to recruit surrounding cells to the implant and to induce adequate differentiation in order to regenerate a natural tendon-to-bone transition. To avoid the rapid removal and degradation of the growth factors after implantation, the loading and binding of the protein on the fiber surface is an important step, which can be realized, e.g., by encapsulation into nanoparticles. This supports the transport and attachment of the proteins to the fiber surface and to tailor release kinetics [22,23]. In the present study, nanoparticles formed from chitosan (CS) and sodium tripolyphosphate (TPP) by ionotropic gelation serve as a loading system for TGF-β_3_. After the loading process, the nanoparticles transform into a layer coating on the fiber surface [24].

In preceding studies, we were able to demonstrate the cytocompatibility (evidenced by a survival rate of over 70%) of CS/TPP nanoparticles for human MSCs up to a concentration of to 625 μg/mL for surface-modified fiber mats and the successful release of TGF-β_3_ from CS/TPP nanoparticles. MSCs subjected to in vitro chondrogenic differentiation with a release medium containing TGF-β_3_ experienced three-dimensional pellet growth, generation of extracellular matrix, and chondrogenic marker gene expression [22,25]. However, these data do not allow the extrapolation of TGF-β_3_ release nor the confirmation of its biological activity when such nanoparticles are attached to a solid carrier material as a film. Therefore, in the present work, we focused on a range of loading concentrations of TGF-β_3_ in the nanoparticle suspension, determined the release from modified PCL fiber mats and studied the chondrogenic differentiation behavior of MSCs in vitro. With regard to the intended future in vivo tests of the implant prototype in small animals, we aimed to release a total dose of 125 ng TGF-β_3_ (minimal effective dose for small animal testing, MED_in vivo_) according to the doses described in the literature (100–150 ng for a single subcutaneous administration to induce chondrogenesis) [26,27].

## 2. Materials and Methods

All chemicals were purchased from Sigma-Aldrich, Taufkirchen, Germany, unless otherwise stated. Human recombinant TGF-β_3_, derived from E. coli, was obtained from PeproTech, Hamburg, Germany. CS, with a degree of acetylation (DA) of 42%, was provided by the Institute for Technical Chemistry, Technische Universität Braunschweig, Braunschweig, Germany, and was produced by acetylation [28,29] of CS (M_n_ 190,000–310,000 g/mol) with a DA of 15-25%, purchased from Sigma-Aldrich. Sodium azide was purchased from Carl Roth, Darmstadt, Germany.

### 2.1. Implant Prototypes

The PCL fiber mats were provided by the Institute of Multiphase Processes, Leibniz Universität Hannover, Hannover, Germany. They were produced as described previously in an optimized electro-spinning process for non-aligned, randomly oriented fibers from PCL with a mean molecular weight of 80,000 g mol^−1^ dissolved with 17% in 2,2,2-trifluoroethanol [30].

After the electro-spinning process, the fiber mat surface was modified with a chitosan-grafted PCL (CS-g-PCL), and, subsequently, with alginate (called “modified fiber mat”), at the Institute for Technical Chemistry, Technische Universität Braunschweig, Braunschweig, Germany, as described by de Cassan et al. [22]. In short, a solution of a graft copolymer consisting of a chitosan backbone and poly(ε-caprolactone) sidechains was used to coat the fibers in order to achieve a positive surface charge. An additional coating from an alginate solution generated a negative charge.

These fiber mats were cut into pieces 16 × 8 mm in size, weighed with a microbalance (XS3DU, Mettler Toledo, Greifensee, Switzerland; for further information, see Section 2.5) and packed into sterilization pouches. The samples were electron-beam-sterilized with a radiation dose of 25 kGy by Mediscan, Kremsmünster, Austria.

### 2.2. Nanoparticle Preparation and Protein Loading

CS with a DA of 42% was added to 0.1% (*v/v*) acetic acid to a concentration of 1 mg/mL and stirred overnight at room temperature to ensure complete dissolution. A solution of TPP at 1 mg/mL was prepared in deionized water.

All of the following steps were executed in a biosafety cabinet under aseptic conditions. Before use, both, the CS and TPP solutions and deionized water were sterile-filtered with a 0.22 µm polyether sulfone filter. TGF-β_3_ was prepared by reconstitution of the lyophilized substance in sterile 10 mM citric acid to a concentration of 400 µg/mL. The required amount of protein was diluted with sterile 10 mM citric acid to the targeted concentration immediately before use.

**Figure 1:**
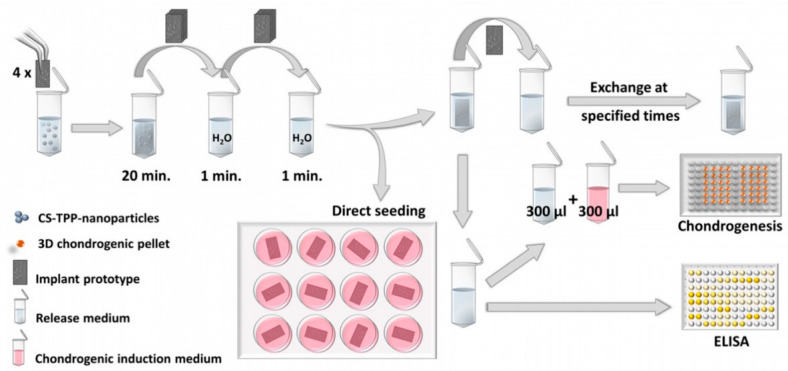
Procedure of fiber mat loading and release experiments for ELISA/cell culture testing. During release experiments, loaded fiber mat samples were transferred to fresh release medium at each sampling time point. Furthermore, the release samples were used to determine the TGF-β_3_ concentration by ELISA and to examine the chondrogenic effect in the cell culture.

Polypropylene low-binding tubes 2 mL in volume (Sorenson BioScience, Salt Lake City, UT, USA) were used for all following steps to avoid protein adsorption. Nanoparticle suspensions were prepared according to Roger et al. [25] by premixing 675 µL of CS solution with 100 µL of TGF-β_3_ solutions to obtain final TGF-β_3_ loading concentrations of 1, 2, 10 and 20 µg/mL. Subsequently, 225 µL of TPP solution was added and rapidly mixed by pipetting to form the nanoparticles within the loading suspension. A stack of four fiber mat samples was placed in each loading suspension and incubated for 20 min at room temperature. After repeated rinsing with 1 mL deionized water for 1 min (Figure 1), the fiber mat samples were separated into individual tubes and left to dry overnight at room temperature in the open tubes within the biosafety cabinet.

### 2.3. Particle Size and Zeta Potential Measurements

Particle sizes and zeta potentials were determined with the Zetasizer Nano ZS (Malvern Instruments, Worcestershire, United Kingdom). Freshly prepared CS/TPP-nanosuspensions, which had been produced in the same manner as for the loading of the fiber mats, were used without further dilution. The determination of the particle sizes was performed at 25 °C with an equilibration time of 180 s and 3 serial measurements with a duration of 300 s each. For zeta potential measurements, a disposable folded capillary cell, an equilibration time of 180 s and 30 runs in each measurement in a series of 3 were used. The results are given as the hydrodynamic diameters (z-average), polydispersity indices (PDI) and zeta potentials of the nanoparticles, as well as the standard deviation of the three serial measurements.

### 2.4. Release Experiments

The release experiments (4 fiber mat samples per loading concentration) with ELISA determination of the released TGF-β_3_ were performed in 2 mL polypropylene low-binding tubes (Sorenson BioScience, Salt Lake City, UT, USA) as well. The release medium was a mixture of phosphate-buffered saline (PBS, Peprotech composition), 0.1% (*w*/*v*) bovine serum albumin (BSA, heat shock fraction, pH 7, ≥98%) and 0.02% (*w*/*v*) sodium azide. The samples were placed individually in 1 mL release medium, and the sealed tubes were put into a climatic chamber at 37 °C. After 1 h, 8 h and 25 h, as well as after 4, 8, 13, 18, 22, 27 and 32 days, the release medium was replaced with fresh release medium (Figure 1). At each medium change, the fiber mat sample was removed from the medium and placed on a KIMTECH precision wipe to thoroughly soak off any embedded medium, thus avoiding carryover of already released protein into the fresh release medium.

A further, slightly modified release experiment was used to prepare the release samples for use in the cell culture (Figure 1, see Section 2.6 and following sections for detailed information about cell culture methods). This experiment was performed with sodium azide-free medium under aseptic conditions, as sodium azide is toxic for cells. This time, the sampling time points were synchronized with the exchange time points of the cell culture medium (a total of 200 µL per well). During each medium change, from 200 µL of spent medium, 100 µL was removed and replaced by 50 µL release medium sample plus 50 µL fresh culture medium [25]. The loading concentrations studied were 0, 1, 10 and 20 µg/mL. Nanoparticles without added TGF-β_3_ (0 µg/mL) were used for two different culture conditions, once with and once without the external addition of 100 µL of 10 ng/mL TGF-β_3_ solution (1 ng TGF-β_3_ during each medium exchange), as positive and negative controls. After 27 days, the cells were examined. In the meantime, all resulting release samples were stored at −20 °C until examination by ELISA. To quantify the exact amount of released TGF-β_3_ in the sodium azide-free release samples for the cell culture, the remainder of the release samples were analyzed via ELISA as well.

### 2.5. ELISA and Data Processing

The ELISA was performed with the Human TGF-beta 3 DuoSet ELISA kit combined with the Duoset ELISA Ancillary Kit 2, purchased from R&D Systems, Minneapolis, USA. The samples were prepared by diluting them with washing buffer from the R&D kit (PBS + 1% BSA) to an appropriate concentration for ELISA calibration. A continuous dilution series (PBS + 1% BSA), starting at 2000 pg/mL and ending at 1.95 pg/mL, was used for calibration. The proprietary TGF-β_3_ standard from the kit was replaced by protein from the identical vial used for nanoparticle loading to optimize the accuracy of the ELISA. All further steps were carried out according to the manual of the kit. The calibration solutions, the blank buffer and each sample were assessed in duplicate. The mean value of both measurements of each sample was calculated and used for further evaluation.

The masses of the individual fiber mat pieces were used to standardize the measured values to the mean mass of all included samples. Since a full change of medium was performed, the released amounts of protein for each time point were cumulated for the release curves. The release rate was defined as the released amount per day during each interval between medium exchanges.

Different fiber mat batches were used for the different experiments. Therefore, the mean fiber diameter (determined via scanning electron microscopy by the Institute of Multiphase Processes, Leibniz Universität Hannover) was used to calculate the mass-specific surface area of each batch, as described by Sundermann et al. [31]. This step served to normalize the release data, since the loaded and consequently released amount of TGF-β_3_ depends on the available surface area.

### 2.6. Cultivation of Bone Marrow-Derived Mesenchymal Stromal Cells

Bone marrow-derived mesenchymal stromal cells (MSCs) from two different donors (A and B, respectively) known to successfully differentiate into the chondrogenic lineage [32] were cultivated in tissue culture flasks at 37 °C and 5% CO_2_ in growth medium (DMEM FG0415 from Biochrom; 10% FCS Hyclone from Thermo Fisher Scientific; 25 mM N-(2-Hydroxyethyl)piperazine-N’-(2-ethanesulfonic acid (HEPES) from Biochrom; 100 U/mL penicillin and 100 µg/mL streptomycin from Biochrom; 2 ng/mL recombinant human FGF-2 from Peprotech) until they reached 70–80% confluency. Cells were detached with trypsin/EDTA (Biochrom) and used for individual experiments with specific cell densities.

### 2.7. Chondrogenic Differentiation

The biological activity of TGF-β_3_ released from modified PCL fiber mats was analyzed with MSCs in a 3D chondrogenic cell pellet culture, as well as by seeding the cells directly onto the fiber mats. For 3D chondrogenesis, the required amount of cells (125,000 cells per pellet) was placed in a conical centrifuge tube and centrifuged at 200× *g* for 5 min. After a washing step with medium containing 4.5 g/L glucose (Biochrom FG 0435) supplemented with 20 mM HEPES from Biochrom, 100 U/mL penicillin and 100 µg/mL streptomycin at 200× *g* for 5 min, the pellet was mixed with the required amount of FG0435 DMEM plus supplements (Biochrom; 20 mM HEPES Biochrom; 100 U/mL penicillin and 100 μg/mL streptomycin; 0.1 μM dexamethasone; 1:100 ITS + supplements (354352, Corning, contains: insulin, human transferrin and selenous acid); 170 μM ascorbate-2-phosphate; 1 mM sodium pyruvate; 350 μM proline). Aliquots of 200 µL of the resulting cell suspension (containing 125,000 cells) were transferred into individual wells of a 96-well plate with round bottoms (polypropylene, 3879, Corning: does not allow cell attachment, but aggregation of the cells into pellets) and centrifuged at 200× *g* for 5 min. Medium exchange was performed after 24 h. Here, the used media (100 µL) were replaced with either fresh media plus release solution (Figure 1) or with media plus PBS/0.1% (*w*/*v*) BSA solution at a 1+1 ratio [25]. For the positive controls, 10 ng/mL soluble recombinant human TGF-β_3_ was used, and the negative controls consisted of media with PBS/0.1% BSA in a 1+1 ratio without TGF-β_3_. Half of the used medium was removed and substituted with freshly mixed medium (as described above) every second day over a time period of 27 days.

The analysis regarding whether cells were able to grow and differentiate on modified fiber mats loaded with TGF-β_3_ was conducted as follows. After the modified fiber mats had been loaded with TGF-β_3_ (see Section 2.2), the mats were washed twice by incubation with PBS at 37 °C and 5% CO_2_ for 30 min. Subsequently they were incubated with the medium at 37 °C and 5% CO_2_ for 60 min in order to prepare the mats for cell seeding. The medium was removed just before starting the experiment. The cells were detached with trypsin/EDTA, and 125,000 cells per mat were seeded onto the fiber mats in a 12-well plate with 1 mL chondrogenic medium. Half of the used medium was removed and substituted with fresh medium twice a week for 27 days.

### 2.8. GAG/DNA

The ratio of glycosaminoglycans (GAG) to DNA was determined by digestion of chondrogenic cell pellets at 60 °C with papain overnight. Subsequently, the DNA was stained with Hoechst 33258 (0.2 µg/mL, Sigma), and the fluorescence was determined with a plate reader (excitation: 360 nm, emission: 460 nm). In order to determine the amount of DNA, a standard series (0, 1, 2, 4, 8, 12, 16, 18, 20 µg/mL) was used. The glycosaminoglycan content of the same digested cell pellet was measured by staining with 1,9-dimethyl methylene blue (0.1 mg/mL, Sigma-Aldrich), and the absorbance was read with a plate reader (absorbance: OD530). In order to determine the GAG amount, a standard series of chondroitin sulfate (5, 12, 25, 50, 75, 100, 150, 200, 300, 400, 500, 600, 800 and 1000 µg/mL) was used. The calculation of the GAG/DNA ratio was performed using Excel.

### 2.9. RNA Isolation and cDNA Synthesis

A homogenizer from bertin technologies (Precellys^®^ 24 lysis and homogenization; 2 × 30 s, 6000 rpm with 40 s pause in between) and a Precellys^®^ RNA Kit (732-3122, VWR) were used to isolate RNA from the chondrogenic cell pellets. The isolation was performed according to the manufacturer’s manual. In short, chondrogenic pellets were washed with PBS and transferred into a tube with ceramic beads in different sizes and 100 µL lysis buffer. After the use of the homogenizer (2 × 30 s, 6000 rpm with 40 s pause in between), an additional 350 µL of lysis buffer was added to the vial and incubated at room temperature for 15 min, with shaking every few minutes. The mixture was transferred into a DNA-removing column and the flow-through was mixed with 70% ethanol before transferring it to the RNA collecting column. The RNA on the membrane was washed twice with washing buffer and eluted with RNase-free water.

In order to remove contamination with genomic DNA, 200 ng RNA was treated with DNase I for 30 min at 37 °C, and, subsequently, cDNA synthesis was performed according to the manufacturer’s manual (Fermentas) using oligo-dT_15_ primer.

### 2.10. qRT-PCR

The Applied Biosystems StepOnePlus instrument (Life Technologies) was used to perform quantitative real-time PCR. According to the manufacturer’s instructions, the analysis was performed using the following gene assays purchased from Life Technologies: RPS29 Hs03004310_g1 (housekeeping gene), SOX9 Hs00165814_m1, COL2A1 Hs01064869_m1, ACAN Hs00153936_m1 and MMP13 Hs00233992_m1.

## 3. Results

### 3.1. Particle Size and Zeta Potential of CS/TPP Nanoparticles

The method used to load the fiber mats with TGF-β_3_ depends on the diffusion behavior and the electric charges of the nanoparticles. Alterations to these parameters might affect the loading process. Therefore, the possible impact of the TGF-β_3_ concentration on CS/TPP-nanoparticle properties and the particle size characteristics (z-average diameter and PDI), as well as their zeta potential, was determined (Table 1). Similar values were obtained for all TGF-β_3_ concentrations as well as for nanoparticles formed in the absence of the protein, and there were no trends in the values depending on the TGF-β_3_ loading concentration. This may indicate unaltered diffusion and electrostatic properties of the tested samples. Slight differences between the values might be related to the manual preparation procedure of the CS/TPP nanoparticles. Furthermore, the zeta potentials were positive for all nanoparticle suspensions; thus, a later interaction with the negatively charged alginate layer on the fibers could be expected.

### 3.2. TGF-β_3_ Release from Implant Samples

**Figure 2:**
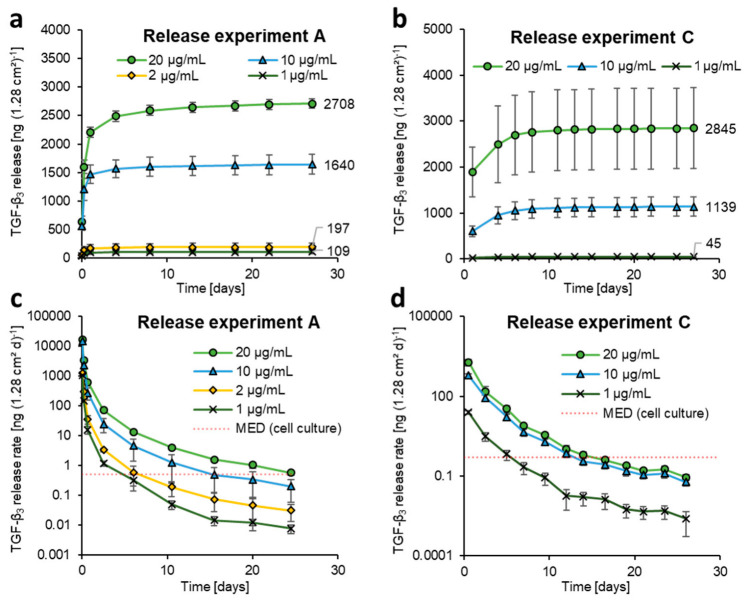
Cumulative release and release rate of TGF-β_3_ from fiber mats (1.28 cm^2^) loaded with various protein concentrations. The graphs show the results of (**a**) release experiment “A” (ELISA analysis) and (**b**) the corresponding release experiment “C” for cell culture (sodium azide-free medium, ELISA analysis, later use for chondrogenic induction). The cumulated amount of TGF-β_3_ released until the end of the experiment increased with the use of higher loading concentrations, as indicated in the two upper graphs. (**c**) The release rate of TGF-β_3_ from fiber mats (1.28 cm^2^) loaded with various protein concentrations (release experiment “A”) and (**d**) the release rate in the corresponding release experiment “C” for the cell culture. With the use of higher loading concentrations, release doses above the minimal effective dose for cell culture (MED_cell culture_, marked in **c**,**d**) of 0.5 ng/d could be maintained for a longer period of time.

Release studies were performed on the fiber mat samples loaded with different concentrations of TGF-β_3_. In Figure 2a, the cumulative release of the fiber mats is shown for release experiment A over time. It is evident that the higher loading concentrations led to an increase in the released amount of TGF-β_3_. The release mode was a burst release, especially during the first 24 h, regardless of the loading concentration used, which has also been observed in the release profile of TGF-β_3_ from suspended nanoparticles in other studies [24,25]. The majority (approx. 95 to 99%) of the protein was released within the first four to eight days. These findings are supported by release experiments B and C, as they showed an increase in the total release depending on the loading concentrations utilized and the characteristic burst release at the beginning of the release process (Figure 2b and Figure S1a).

For release experiment A, the total release after loading with 2 µg/mL compared to that with 1 µg/mL was nearly doubled, which also applies to the total TGF-β_3_ release values of the fiber mats loaded with 20 and 10 µg/mL. In the case of loading with 10 µg/mL compared to 1 µg/mL or 20 µg/mL compared to 2 µg, the total release was more than ten times higher. This finding might indicate that the loading becomes more efficient with an increasing loading concentration.

In release experiments B (Figure S1a) and C (Figure 2b), the increases in the total released amounts were different from those observed in experiment A. Hence, the differences might, rather, be caused by a deviation due to the small number of samples (n = 4) or by the general increase in variations in total release with the use of higher loading concentrations. In order to establish a connection between the observations made in all release experiments, the values of total TGF-β_3_ release for the included individual samples were plotted against the corresponding loading concentration. The resulting graph is discussed in Section 3.3.

### 3.3. Correlation between TGF-β_3_ Loading Concentration and Total Release

**Figure 3:**
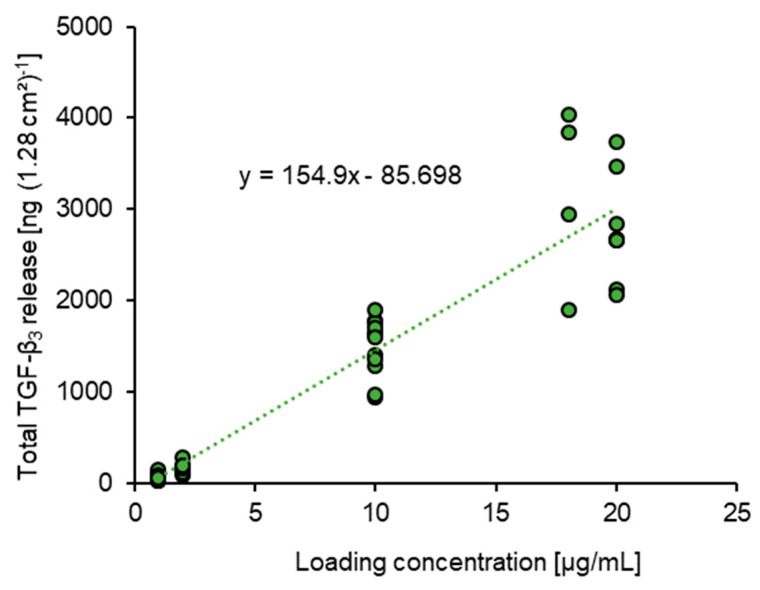
Correlation between TGF-β_3_ loading concentration and total release from implant prototype. The adjusted coefficient of determination for a linear correlation was 0.881, while variations increased with higher loading concentrations.

To gain a better understanding of the correlation between loading concentration and the resulting total release amount of TGF-β_3_, all corresponding data were combined into a single graph (Figure 3). In spite of the considerable variability, particularly for the data obtained from fiber mats loaded with high TGF-β_3_ concentrations, the linear correlation between both parameters was obvious. The low adjusted coefficient of determination for a linear fit of 0.881 was presumably due to the rather high variation in samples loaded with higher concentrations of TGF-β_3_. The linear function of the fit may be useful to approximately estimate the total release to be expected at a given loading concentration. Inversely, this function could serve to choose a suitable loading concentration for the desired application.

### 3.4. Evolution of TGF-β_3_ Release Rate over Time

To induce chondrogenesis in cell culture, a minimal effective dose (MED_cell culture_) of 0.5 ng per day over a minimum time period of 14 days is required (Figure S3). To determine the loading concentration required for this purpose, in Figure 2c,d the release rate over time is shown, as well as the threshold for chondrogenesis. In all three release studies (A, Figure 2c; B, Figure S1b; C, Figure 2d) the minimum release rate of 0.5 ng/day over at least 14 days was achieved with 20 µg/mL TGF-β_3_ loading concentration. In release experiment A, the loading with 10 µg/mL also met this aim, while in the other experiments, this loading concentration resulted in a release rate that dropped below 0.5 ng/d too quickly.

### 3.5. Calculation of Expected In Vivo Dose

Since the studies also served to prepare for in vivo experiments, we used the results to calculate the expected total release of smaller fiber mat samples. For testing in small animals, the implant prototypes should be 5 × 3 mm = 15 mm^2^, and the mats used in the present studies measured 16 × 8 mm = 128 mm^2^. Thus, the total release is expected to be 8.53 times lower from fiber mat prototypes for the intended in vivo applications. The resulting doses for loading concentrations of 1, 2, 10 and 20 µg/mL, calculated from the linear correlation function established in Section 3.3 for 128 mm^2^ fiber mats, and the doses for 15 mm^2^ fiber mats, calculated via the dimension factor of 8.53, are listed in Table 2, including a score denoting whether the requirements would be met. According to these calculations, the MED_in vivo_ for animal testing (125 ng [26,27]) would be met by using loading concentrations of 10 or 20 µg/mL for 15 mm^2^ fiber mats. The results of all release experiments performed (A, Figure 2a; B, Figure S1a; C, Figure 2b), support this assumption. Furthermore, the correlation function results in a minimum loading concentration of 7.44 µg/mL TGF-β_3_, for a total release of 125 ng from the 15 mm^2^ fiber mats.

### 3.6. Chondrogenic Differentiation

**Figure 4:**
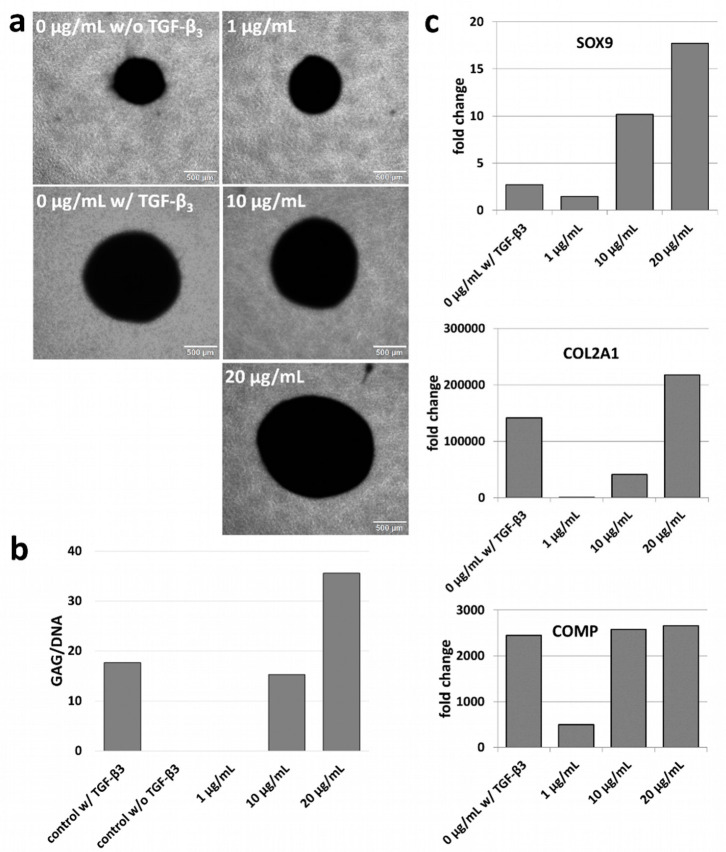
Chondrogenic differentiation of MSCs from donor A was enhanced with increasing loading concentrations. w/: with TGF-β_3_, w/o: without TGF-β_3_. (**a**) Chondrogenic cell pellets. In the presence of TGF-β_3_ (0 µg/mL w/ TGF-β_3_ (i.e., release medium of fiber mats w/o TGF-β_3_ loading, but with external addition of TGF-β_3_, positive control), 1 µg/mL, 10 µg/mL, 20 µg/mL), the morphology of the pellets was round and compact, and the size increased with increasing loading concentrations. In contrast, without TGF-β_3_ (0 µg/mL w/o TGF-β_3_), the pellets showed considerably less compact growth and were smaller in size. Scale bar: 500 µm. (**b**) Ratio of glycosaminoglycan (GAG) to DNA. The increasing loading concentration led to an increase in GAG/DNA. Cell pellets, which were not treated with TGF-β_3_ (0 µg/mL w/o TGF-β_3_) or were treated with release medium from the lowest loading concentration (1 µg/mL), showed a very low GAG/DNA ratio. (**c**) Gene expression analysis of the chondrogenic marker genes *SOX9*, *COL2A1* and *COMP*. Shown is the fold change against 0 µg/mL w/o TGF-β_3_. All three chondrogenic marker genes resulted in an increase in expression with the treatment of TGF-β_3_, and almost no expression without TGF-β_3_ treatment.

Given the successful increase in TGF-β_3_ released from fiber mats loaded with higher loading concentrations, a corresponding enhancement of the chondrogenic differentiation behavior of MSCs was studied. Initially, analyses in 3D cell pellet setups, a well-recognized method for chondrogenic in vitro differentiation, were performed, and the pellets were cultivated with a mixture of release medium and cell culture medium (Figure 4). After a cultivation period of 27 days with MSCs from donor A, the pellets incubated with externally added soluble TGF-β_3_ (Figure 4a; 0 µg/mL w/ TGF-β_3_) showed a notable increase in size in comparison to the negative control without TGF-β_3_ (Figure 4a; 0 µg/mL w/o TGF-β_3_). The pellet size increased with the increasing loading concentrations of TGF-β_3_ used in the release experiments. The pellet incubated with the release medium of the lowest loading concentration (1 µg/mL) was smaller than the one incubated with 10 µg/mL TGF-β_3_, and an even larger pellet was observed with the highest loading concentration (20 µg/mL, Figure 4a, right). The same could be observed for MSCs from donor B (Figure S2a). This effect correlated with formation of extracellular matrix, since larger pellets demonstrated a higher GAG-to-DNA ratio. Cell pellets, which were not treated with TGF-β_3_ at all (0 µg/mL w/o TGF-β_3_) or were treated only with release medium resulting from the lowest loading concentration (1 µg/mL), showed a very low GAG/DNA ratio (Figure 4b). Gene expression analysis of chondrogenic marker genes resulted in a similar tendency. The negative controls without TGF-β_3_ showed almost no expression of *SOX9*, *COL2A1* or *COMP* and relatively low expression for 1 µg/mL, but this increased with increasing loading concentrations or with the addition of external TGF-β_3_ (Figure 4c).

**Figure 5:**
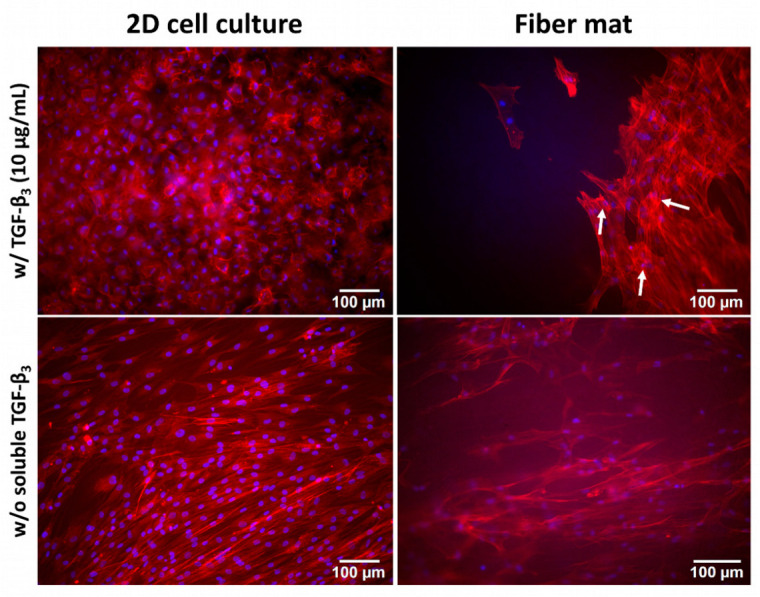
MSCs seeded as positive and negative controls in a 2D manner (**left panel**) as well as on modified fiber mats loaded with empty chitosan nanoparticles (**right panel**). Cells were stained with phalloidin (red) and DAPI (blue) to visualize the actin cytoskeleton and the nuclei, respectively. Both sets were analyzed in the presence and absence of TGF-β_3_ (upper and lower panel, respectively). The control with TGF-β_3_ under 2D conditions showed rounded cells (**left**, upper panel), with morphology similar to that of chondrocytes and in striking contrast to the elongated fibroblast-like cells observed in the absence of TGF-β_3_ (**left**, lower panel). The right panel shows MSCs seeded on modified fiber mats loaded with empty chitosan nanoparticles. The right upper panel shows the condition with TGF-β_3_ with some rounded cells (white arrow) and the lower panel without TGF-β_3_.

The next step was the direct seeding of MSCs onto the modified fiber mats loaded with different concentrations of TGF-β_3_. Here, we analyzed two positive and two negative sets of controls. For Set 1, the cells were seeded on cell culture plates (2D) both with (control w/ TGF-β_3_) and without the addition of external TGF-β_3_ (control w/o TGF-β_3_). For Set 2, the cells were seeded on modified fiber mats loaded with empty chitosan nanoparticles both with and without the addition of external TGF-β_3_ (Figure 5). A difference in cell morphology in the absence and presence of TGF-β_3_ was observed. The positive 2D control showed a round morphology in comparison to the negative control, reminiscent of the morphology of chondrocytes (Figure 5, left). The cells seeded on fiber mats exhibited a slightly different phenotype. The addition of TGF-β_3_ caused cell growth in several layers, and some cells began to develop a round morphology (Figure 5, right upper panel, white arrows), whereas the cells without TGF-β_3_ showed the elongated, fibroblast-like morphology of MSCs (Figure 5, right lower panel).

In summary, the addition of TGF-β_3_ caused cells seeded onto modified fiber mats or onto cell culture plates to develop a chondrocyte-like morphology.

**Figure 6:**
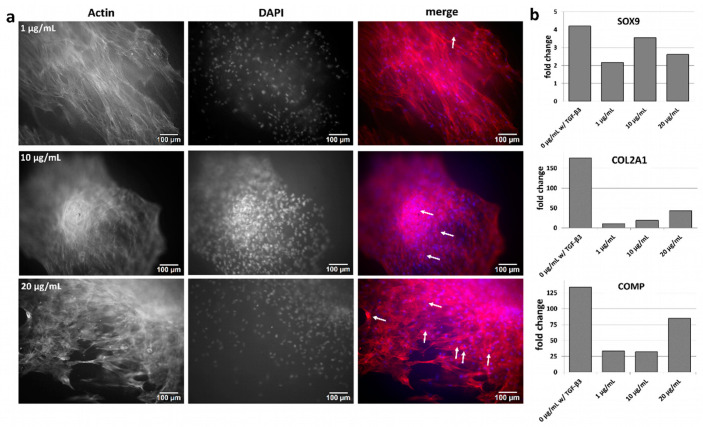
Chondrogenic differentiation on modified fiber mats. (**a**) Modified fiber mats loaded with different TGF-β_3_ concentrations (1 µg/mL (**upper panel**), 10 µg/mL (**middle panel**) and 20 µg/mL (**lower panel**)), were seeded with MSCs and cultivated for 27 days. Cells were stained with phalloidin for the actin cytoskeleton and with DAPI for the DNA. Chondrocyte-like cells are marked with white arrows in the merge panel. Scale bar: 100 µm. (**b**) Gene expression analyses of chondrogenic marker genes *SOX9*, *COL2A1* and *COMP*, normalized to the housekeeper *RPS29* and shown in fold change to the negative control 0 µg/mL w/o TGF-β_3_.

Furthermore, it was investigated whether the modified fiber mats loaded with different TGF-β_3_ concentrations showed similar effects. Here, the modified fiber mats were loaded with different TGF-β_3_ concentrations (1, 10 and 20 µg/mL). Cells were seeded directly onto the loaded fiber mats and analyzed after 27 days for morphology and gene expression. With increasing loading concentration, more cells developed a round morphology. The concentration of 1 µg/mL TGF-β_3_ led to just a few rounded cells (Figure 6a, white arrow, upper right panel, “merge”), while the concentration of 10 µg/mL TGF-β_3_ resulted into more rounded cells (Figure 6a, white arrows, middle right panel, “merge”). Even more rounded cells were observed at a 20 µg/mL TGF-β_3_ loading concentration (Figure 6a, white arrows, lower right panel, “merge”).

In the gene expression analyses, the chondrogenic marker genes *SOX9*, *COL2A1* and *COMP* were almost undetectable for the negative controls without the external addition of TGF-β_3_, and a very low expression was observed for the 1 µg/mL condition. However, with higher loading concentrations, the gene expressions of *COL2A1* and *COMP* increased while *SOX9* demonstrated low induction, which was at its highest with loading concentration of 10 µg/mL (Figure 6b).

## 4. Discussion

The regeneration of bone, cartilage and tendon tissue is of increasing relevance in the medical field. In the present study, different concentrations of TGF-β_3_ were loaded onto modified PCL fiber mats and were able to successfully release a required amount of protein from these fiber mats.

Our aim was to deliver a minimum dose of 125 ng per fiber mat piece measuring 5 × 3 mm, that are the dimensions expected to be suitable for studies in small animals. With the use of loading concentrations from 1 to 20 µg/mL of TGF-β_3_, we achieved a wide range of total released doses, including amounts high enough for the envisaged in vivo experiments in mice and/or rats. Furthermore, we were able to show that the daily MED_cell culture_ for TGF-β_3_ of 0.5 ng for a time period of 14 days could be delivered with implant prototypes loaded with 20 µg/mL TGF-β_3_. Hence, chondrogenesis was expected to occur in cell culture studies with this loading concentration.

Loading concentrations and total release of TGF-β_3_ showed a clear correlation. With a linear fitting trend-line function, we were able to approximately predict the anticipated release for a fixed loading concentration. Vice versa, the function could serve as a tool to determine the required loading concentration for a desired release dose.

While ELISA confirmed the successful release of TGF-β_3_ in our study, this does not necessarily mean that the released protein was still biologically active. Protein activity cannot be taken for granted after encapsulation in nanoparticles, the loading process and the release. Therefore, as an important complementary addition, cell culture tests confirmed that TGF-β_3_ was released in a biologically active form. The release of TGF-β_3_ from the fiber mats loaded with high concentrations enhanced the chondrogenic differentiation considerably, especially for the 20 µg/mL loading concentration. With increasing loading concentrations, an increase in the chondrogenic responses of the cells was clearly documented. Depending on the TGF-β_3_ concentration used for nanoparticle loading, at the time of evaluation (after 27 days for all treatments), the cells were in different stages of chondrogenesis. A low TGF-β_3_ loading concentration and, thus, a low total release led to early stages of chondrogenic differentiation along with expression of early chondrogenic marker genes, exemplified by *SOX9*, a low GAG/DNA ratio and the small pellet sizes. With increasingly higher loading concentrations, expressions of late chondrogenic marker genes (*COL2A1*, *ACAN*) could be detected, the GAG/DNA ratio became higher and the cell pellets grew in size.

Sundermann et al. were able to prepare an implant based on an electrospun and surface-modified (CS-g-PCL and alginate coating) PCL carrier. In that study, CS/TPP-nanoparticles were also used to load BMP-2 and TGF-β_3_ onto the fiber surfaces in separate areas of the implants. The spatial gradation of the two loaded growth factors was meant to induce stem cell differentiation in a graded manner, matching the physiological tissue transition between bone and cartilage in entheses [23]. In these studies, successful loading and release of BMP-2 and TGF-β_3_ were achieved in vitro. However, for future in vivo applications, the total release doses were too low, and they would require adaptation [26,27,33]. With the present work, we solved the first of these challenges by enabling the adequate and bioactive release of TGF-β_3_ for this delivery system.

Various cell-free strategies for the treatment of cartilage injuries have already been tested in vivo. Scaffolds obtained by the decellularization of cartilage or bone tissue, from other naturally-derived materials such as collagens, as well as synthetically produced scaffolds with or without additional loading of TGF-β_3_, successfully recruited mononuclear cells, including stem cells, and thus improved the regeneration of tissue in artificial defects [34,35,36,37,38]. Following these examples, in vivo studies would now be appropriate to further demonstrate the applicability of the system presented in the current study.

The transforming growth factor β isoforms are known for their importance in wound healing and tissue regeneration, as well as for their potential impact on the formation of fibrotic tissue [39,40,41]. Further, it has been found that inhibition of TGF-β with a soluble TGF-β type II receptor reduced fibrotic effects depending on the administered dose [42]. Another work indicated a dose-dependency for agents inducing the TGF-β/Smad signaling pathway, mediating kidney fibrosis [42,43]. Hence, this pro-fibrotic effect may possibly be dose-dependent in peripheral tissue as well, and would need to be reconsidered if the current approach for dose adaption was transferred to in vivo applications.

## 5. Conclusions

The present examinations and findings contribute to the development of scalability between in vitro and in vivo use of regenerative implants for tissue transitions based on the controlled release of biologically active factors from nanofiber carriers. Besides the general demonstration of successful release of TGF-β_3_ from electrospun PCL fiber mats, we were able to prove the biological activity of the released growth factor via a dose-dependent chondrogenic response in MSCs. Thereby, a sound basis for in vivo studies was created. The correlation between the loading concentration and total release could be used to support the preparation of implant prototypes with defined release doses, which may be helpful in adjusting the implant for different applications.

The next important step to prove the transferability needs to be actual in vivo analyses in small animals. Through these studies, it would become possible to gain a better understanding of the distribution, elimination processes and defense reactions of the organism, such as inflammation or fibrosis after implantation.

## Figures and Tables

**Table 1:** Size, PDI and zeta potential of CS/TPP nanoparticles loaded with different TGF-β_3_ concentrations.

TGF-β_3_ Concentration	Z-average Diameter (nm)	PDI	Zeta Potential (mV)
0 µg/mL	267.8 ± 2.38	0.262 ± 0.003	16.2 ± 1.01
1 µg/mL	259.9 ± 1.19	0.258 ± 0.002	16.6 ± 0.75
2 µg/mL	278.3 ± 0.81	0.253 ± 0.006	17.6 ± 0.60
10 µg/mL	264.1 ± 0.87	0.259 ± 0.003	16.9 ± 0.40
20 µg/mL	249.4 ± 0.23	0.252 ± 0.002	16.6 ± 0.58

**Table 2:** Calculated total release of TGF-β_3_ from fiber mats with dimensions used for in vivo studies in small animals (15 mm^2^).

Loading Concentration [µg/mL]	Calculated Total Release from 128 mm^2^ Fiber Mats (ng)	Calculated Total Release from 15 mm^2^ Fiber Mats (ng)	MED_in vivo_ Achieved
20	3012.3	353.0	yes
10	1463.3	171.5	yes
2	224.1	26.3	no
1	69.2	8.1	no

## Data Availability

Data are contained within the article.

## Supplementary Materials

The following supporting information can be downloaded at: https://www.mdpi.com/article/10.3390/pharmaceutics15041303/s1, Figure S1: Cumulative release (a) and release rate (b) of TGF-β_3_ from fiber mats (1.28 cm^2^) loaded with various protein concentrations in a repetitive experiment (“release experiment B”); Figure S2: Chondrogenic differentiation of MSCs from donor B increases with increasing loading concentration; Figure S3: Chondrogenic differentiation after addition of TGF-β_3_ (5 ng/mL) over specific periods.
