# Supplementary material for: Release of TGF-β3 from Surface-Modified PCL Fiber Mats Triggers a Dose-Dependent Chondrogenic Differentiation of Human Mesenchymal Stromal Cells
Source: Pharmaceutics. 2023 Apr 21;15(4):1303. doi: 10.3390/pharmaceutics15041303 (PMC10146193; doi:10.3390/pharmaceutics15041303)
Supplement: Supplementary file 1 [file pharmaceutics-15-01303-s001.zip › pharmaceutics-2313746-supplementary.pdf]

pharmaceutics

MDPI

Supporting Information

Article: Release of TGF-β3 from surface-modified PCL fiber mats triggers a dose-dependent chondrogenic differentiation of human mesenchymal stromal cells

Leonie Berten-Schunk[1], Yvonne Roger[2,3], Heike Bunjes[1,4*], Andrea Hoffmann[2,3*]

[1] Technische Universität Braunschweig, Institut für Pharmazeutische Technologie und Biopharmazie, 38106 Braunschweig, Germany

[2] Hannover Medical School, Department of Orthopedic Surgery, Graded Implants and Regenerative Strategies, Laboratory of Biomechanics and Biomaterials, 30625 Hannover, Germany

[3] Niedersächsisches Zentrum für Biomedizintechnik, Implantatforschung und Entwicklung (NIFE), 30625 Hannover, Germany

[4] Technische Universität Braunschweig, Zentrum für Pharmaverfahrenstechnik (PVZ), 38106 Braunschweig, Germany

Leonie Berten-Schunk and Yvonne Roger contributed equally to this paper.

* Correspondence: heike.bunjes@tu-braunschweig.de , Hoffmann.Andrea@mh-hannover.de

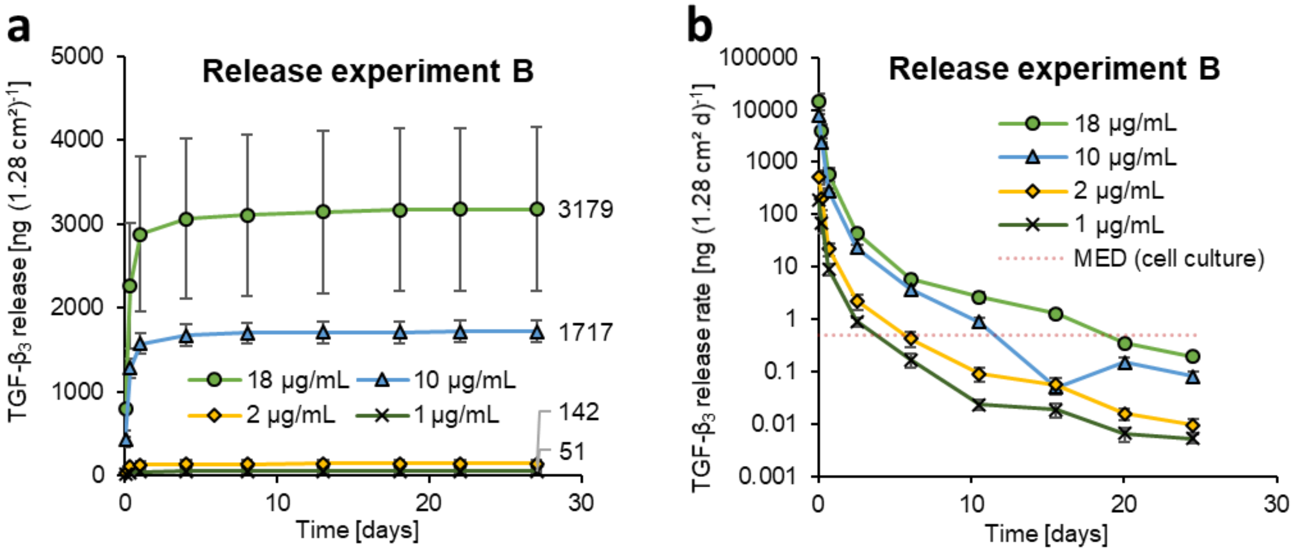

Figure S 1 Cumulative release (a) and release rate (b) of TGF-β_3 from fiber mats (1.28 cm²) loaded with various protein concentrations in a repetitive experiment ("release experiment B").

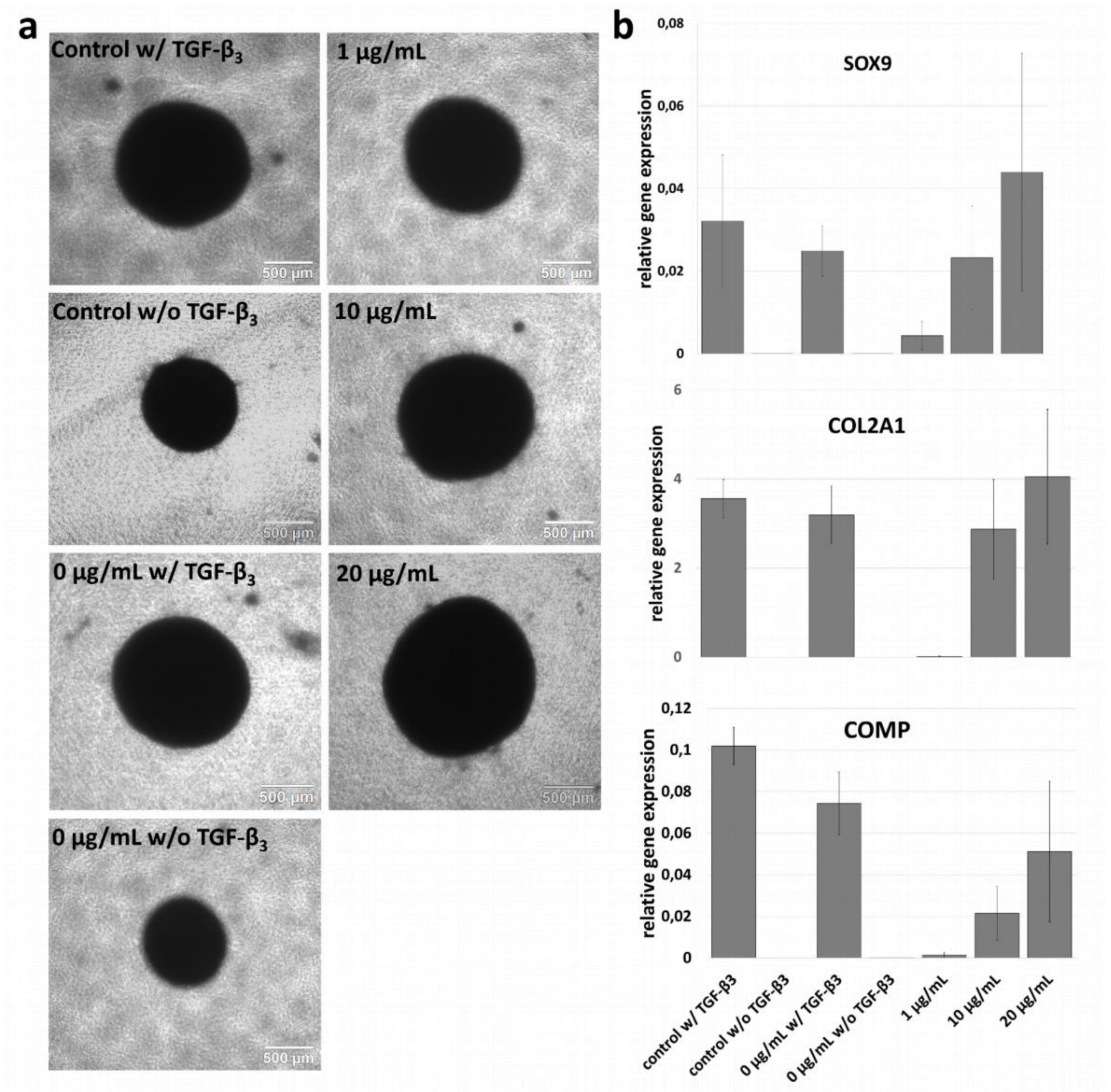

Figure S 2 Chondrogenic differentiation of MSCs from donor B increases with increasing loading concentration. (a) shows chondrogenic cell pellets. In the presence of TGF-β_3 (0 µg/mL w/ TGF-β_3 (a fiber mat without TGF-β_3 loading but external addition of TGF-β_3), 1 µg/mL, 10 µg/mL, 20 µg/mL) the morphology of the pellet is round, compact and the size increases with increasing loading concentration. In contrast, without TGF-β_3 the pellet shows considerably less compact growth and size. Scale bar: 500 µm. (b) demonstrates the gene expression analysis of the chondrogenic marker genes *SOX9*, *COL2A1* and *COMP* (relative gene expression). All three chondrogenic marker genes show an increase upon treatment with TGF-β_3 and almost no expression without TGF-β_3 treatment.

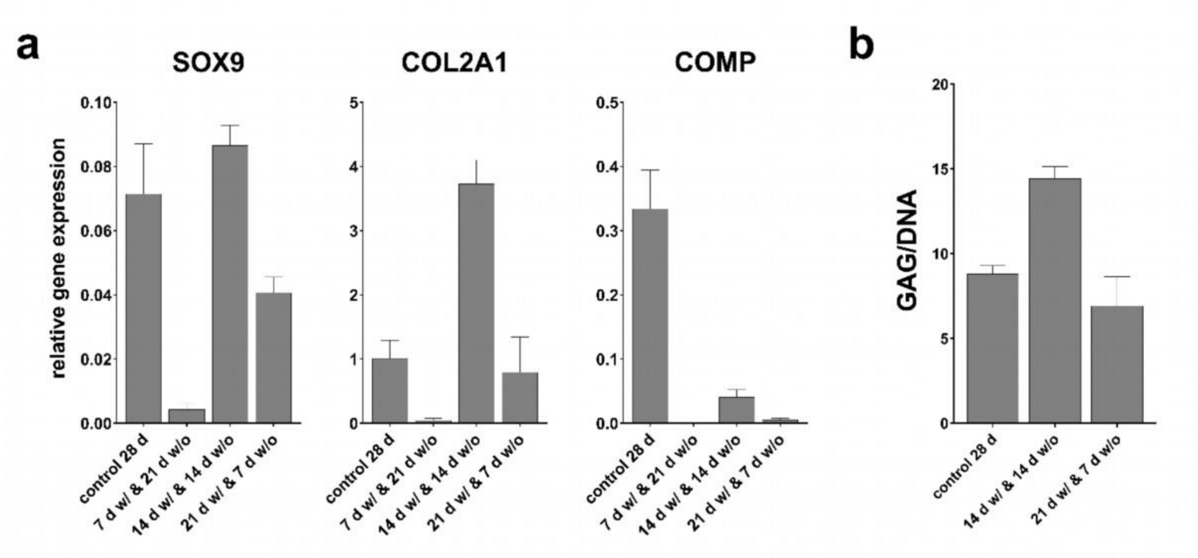

Figure S 3 Chondrogenic differentiation after addition of TGF-β3 (5 ng/mL) over specific periods. (a) Relative gene expression analysis is shown for *SOX9*, *COL2A1* and *COMP* after the addition of TGF-β3 over 28 days (control 28 d), 7 days and further incubation of 21 days without TGF-β3 (7 d w/ & 21 d w/o), 14 days and further incubation of 14 days (14 d w/ & 14 days w/o) and 21 days and 7 days without TGF-β3 (21 d w/ & 7 d w/o). All three chondrogenic marker genes show an increase with the increasing incubation time of TGF-β3, with showing the lowest expression for 7 d w/ & 21 d w/o. (b) GAG/DNA ratio showing highest ratio for 14 days of TGF-β3 incubation and further 14 days without TGF-β3 incubation (14 d w/ & 14 d w/o).
